# Antimicrobial targets localize to the extracellular vesicle-associated proteome of *Pseudomonas aeruginosa* grown in a biofilm

**DOI:** 10.3389/fmicb.2014.00464

**Published:** 2014-09-03

**Authors:** Amber J. Park, Matthew D. Surette, Cezar M. Khursigara

**Affiliations:** Department of Molecular and Cellular Biology, University of GuelphGuelph, ON, Canada

**Keywords:** proteomics, *Pseudomonas aeruginosa*, outer membrane vesicles, antimicrobial resistance, bacterial biofilms

## Abstract

Microbial biofilms are particularly resistant to antimicrobial therapies. These surface-attached communities are protected against host defenses and pharmacotherapy by a self-produced matrix that surrounds and fortifies them. Recent proteomic evidence also suggests that some bacteria, including the opportunistic pathogen *Pseudomonas aeruginosa,* undergo modifications within a biofilm that make them uniquely resistant compared to their planktonic (free-living) counterparts. This study examines 50 proteins in the resistance subproteome of both surface-associated and free-living *P. aeruginosa* PAO1 over three time points. Proteins were grouped into categories based on their roles in antimicrobial: (i) binding, (ii) eﬄux, (iii) resistance, and (iv) susceptibility. In addition, the extracellular outer membrane vesicle-associated proteome is examined and compared between the two growth modes. We show that in whole cells between 12–24% of the proteins are present at significantly different abundance levels over time, with some proteins being unique to a specific growth mode; however, the total abundance levels in the four categories remain consistent. In contrast, marked differences are seen in the protein content of the outer membrane vesicles, which contain a greater number of drug-binding proteins in vesicles purified from late-stage biofilms. These results show how the method of analysis can impact the interpretation of proteomic data (i.e., individual proteins vs. systems), and highlight the advantage of using protein-based methods to identify potential antimicrobial resistance mechanisms in extracellular sample components. Furthermore, this information has the potential to inform the development of specific antipseudomonal therapies that quench possible drug-sequestering vesicle proteins. This strategy could serve as a novel approach for combating the high-level of antimicrobial resistance in *P. aeruginosa* biofilms.

## INTRODUCTION

**FIGURE 1:**
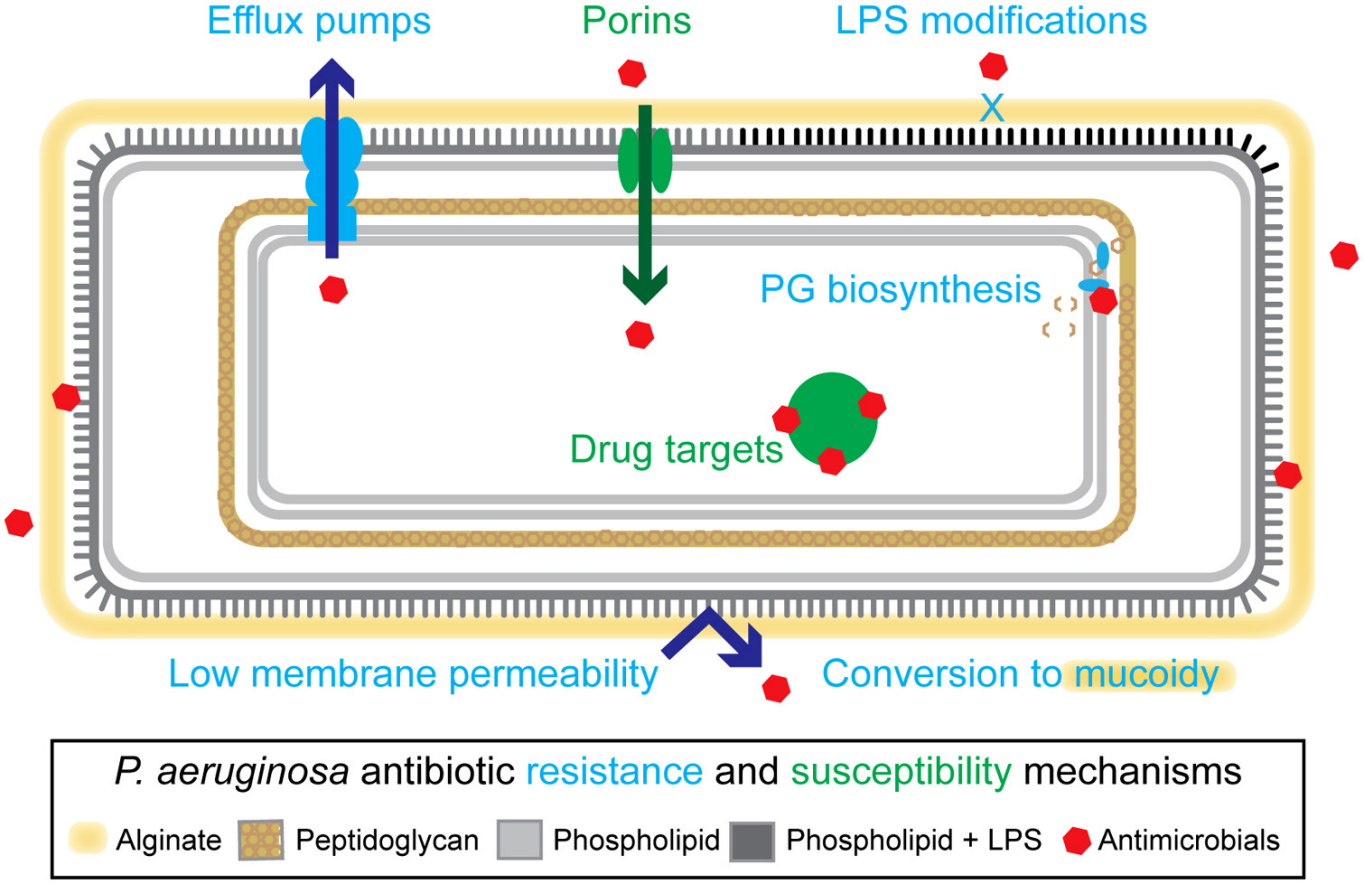
**Overview of resistance mechanisms in *Pseudomonas aeruginosa*.** *P. aeruginosa* have a variety of intrinsic and adaptive resistance mechanisms including: a highly impermeable membrane, conversion to mucoidy, active eﬄux of antimicrobials, reduction of outer membrane porins, lipopolysaccharide (LPS) modifications, and modification of drug targets. Examples of specific proteins involved in these mechanisms include: MexAB-OprM (eﬄux pump), OprD (porin), ArnA and ArnB (LPS modifying proteins), GyrA (drug target, DNA metabolic processes), and NagZ (peptidoglycan-based cell wall biogenesis). See main text for references.

The opportunistic pathogen *Pseudomonas aeruginosa* is a common agent of infectious disease in immunocompromised individuals (Afessa and Green, 2000; Öncül et al., 2013; Chatterjee et al., 2014), and is the dominant pathogen in late-stage cystic fibrosis (CF; Rajan and Saiman, 2002; Rudkjobing et al., 2012). *P. aeruginosa* has many features that make it intrinsically resistant to antimicrobial therapies, including a low membrane permeability (Angus et al., 1982; Yoshimura and Nikaido, 1982), and an extensive collection of multidrug eﬄux pumps (Li et al., 1995, 2003; Poole et al., 1996; Köhler et al., 1997; Morita et al., 2001; Aendekerk et al., 2002; Chuanchuen et al., 2002; Mima et al., 2005, 2007, 2009; **Figure 1**). Additional mechanisms, including lipopolysaccharide modifications (Ernst, 1999; Ernst et al., 2005; Cigana et al., 2009) and conversion to mucoidy (Lam et al., 1980), which are established during adaptation to the CF lung environment, further enhance *P. aeruginosa*’s recalcitrant nature (Nichols et al., 1988; Pritt et al., 2007; Needham and Trent, 2013; **Figure 1**). Ultimately, chronic pulmonary infections in CF become unyielding to treatment, resulting in high levels of patient morbidity and mortality.

During infection, *P. aeruginosa* transitions from an independent, free-swimming lifestyle (i.e., planktonic) into sessile aggregates of bacteria called biofilms. These structures are surrounded by a self-produced extracellular matrix consisting of proteins, DNA and polysaccharides, which acts as a physical and physiological barrier against both host-produced and pharmaceutical antimicrobials. Some of the protection is provided by the physical structure of the extracellular matrix, which can limit penetration (Jefferson et al., 2005) and directly bind some classes of antibiotics (Gordon et al., 1988; Chiang et al., 2013). A second level of protection is provided by the unique physiology of the biofilm matrix, including metabolic and oxygen gradients (Anderl et al., 2003; Walters et al., 2003; Wessel et al., 2014). These gradients reduce the efficacy of drugs that target growth and metabolic processes (Tuomanen et al., 1986; Walters et al., 2003), or are affected by anaerobic conditions (Borriello et al., 2004). In addition, it has been suggested that chromosomally encoded drug-deactivating enzymes may concentrate in the biofilm matrix and decrease the efficacy of certain antimicrobials, such as beta-lactams (Anderl et al., 2000; Bagge et al., 2004; Mulet et al., 2011). Accordingly, experimental systems designed to specifically challenge sessile cells with antimicrobials indicate that *P. aeruginosa* biofilms require inhibitory concentrations that are often orders of magnitude higher than those required for planktonic controls (Ceri et al., 1999).

The question that remains is whether the bacteria themselves undergo specific changes within the biofilm that make them more resistant than their planktonic counterparts. Whiteley et al. (2001) examined gene expression in a *P. aeruginosa* PAO1 biofilm using DNA microarray techniques. Surprisingly, they noted that only 1% of the genes were differentially expressed between planktonic and biofilm cultures, and only a handful of genes had potential roles in antimicrobial resistance (Whiteley et al., 2001). More recently, RNA sequencing (Dötsch et al., 2012) and meta-analysis (Folsom et al., 2010) strategies have investigated if the transcriptomes of biofilm and planktonic *P. aeruginosa* provide any clues to their high level of resistance. These authors generally concluded that at the transcript level biofilms displayed expression patterns indicative of oxygen limitation and slowed metabolism (Folsom et al., 2010; Dötsch et al., 2012). Overall, their expression profiles showed a considerable amount of overlap with stationary phase planktonic cultures. Importantly, no specific resistance mechanisms were identified. Though the lack of findings related to biofilm resistance in these studies is unexpected, it is possible that alternate technical approaches may reveal further insight. Specifically, early studies using mass spectrometry (MS)-based approaches demonstrated qualitative differences in 10–50% of the identified proteins between *P. aeruginosa* biofilms and planktonic cultures at different time points (Sauer et al., 2002; Vilain et al., 2004). While mechanisms of antibiotic resistance were not a focus of these studies, these findings do suggest that biofilm-specific cellular changes may occur at a post-transcriptional level. Furthermore, by directly comparing mRNA and protein levels in the same sample, others have found that (i) the cellular abundance of protein is primarily controlled at the level of translation (Schwanhäusser et al., 2011); (ii) the correlation between mRNA and protein abundance within a single sample type is often low (Nie et al., 2006; Taniguchi et al., 2010; Maier et al., 2011; Walley et al., 2013); and (iii) proteins are more stable and have a greater dynamic range than mRNA (Schwanhäusser et al., 2011).

Despite 10 years worth of studies it is still unclear if specific cellular changes contribute to the increased antibiotic resistance of *P. aeruginosa* growing in a biofilm. However, evidence suggests the examination of the proteomes of planktonic and biofilm cultures will provide the most accurate molecular description of these cells. We recently established a MS-based experimental platform and used spectral counting methods to quantify over 1884 whole cell proteins in planktonic and biofilm cultures of *P. aeruginosa* at three time points during development (Park et al., 2014). This study demonstrated that ~9% of the identified high-quality proteins were either unique to one of the growth modes, or present at significantly different abundance levels. One of the proteins we highlighted in this study was GyrA (PA3168). This protein, which is part of the DNA topoisomerase complex (Winsor et al., 2010), is a target of the quinolone class of antibiotics (Wishart et al., 2007) and was significantly less abundant in biofilm cultures at the 48 h time point (Park et al., 2014).

In this current study we completed a focused examination of a subset of proteins in our dataset with known roles in antibiotic resistance. We used MaxQuant software (Cox and Mann, 2008) for improved identification and quantification of proteins that were chosen based on: (i) a Pseudomonas Community Annotation Project (PCAP) designation of a “Antibiotic resistance and susceptibility” protein (Winsor et al., 2010; further subdivided into two separate categories); (ii) eﬄux proteins with gene ontology (GO; Ashburner et al., 2000) annotations of transporter or porin activity; and (iii) known drug targets (Winsor et al., 2010). In addition, we isolated outer membrane vesicles (OMV) from the cell-free supernatant (CFS) of these cultures and analyzed them using the same MS-based platform and MaxQuant software. These structures have been shown to play a role in biofilm formation (Yonezawa et al., 2009), toxin packaging and delivery (Kesty et al., 2004; Bauman and Kuehn, 2009; Bomberger et al., 2009; Haurat et al., 2011), induction of inflammatory responses (Bauman and Kuehn, 2006; Chatterjee and Chaudhuri, 2013; Jun et al., 2013; Kim et al., 2013; Park et al., 2013; Thay et al., 2013), cell–cell communication (Berleman and Auer, 2012), and bacterial survival (Manning and Kuehn, 2011). With this study we aim to determine the contributions of the whole cell and OMV proteomes to antibiotic resistance in *P. aeruginosa* biofilms, and to identify targets for the development of biofilm-specific antimicrobial therapy.

## RESULTS

### *P. aeruginosa* PAO1 FORMS STRUCTURED BIOFILMS THAT CONTAIN OMVs

**FIGURE 2:**
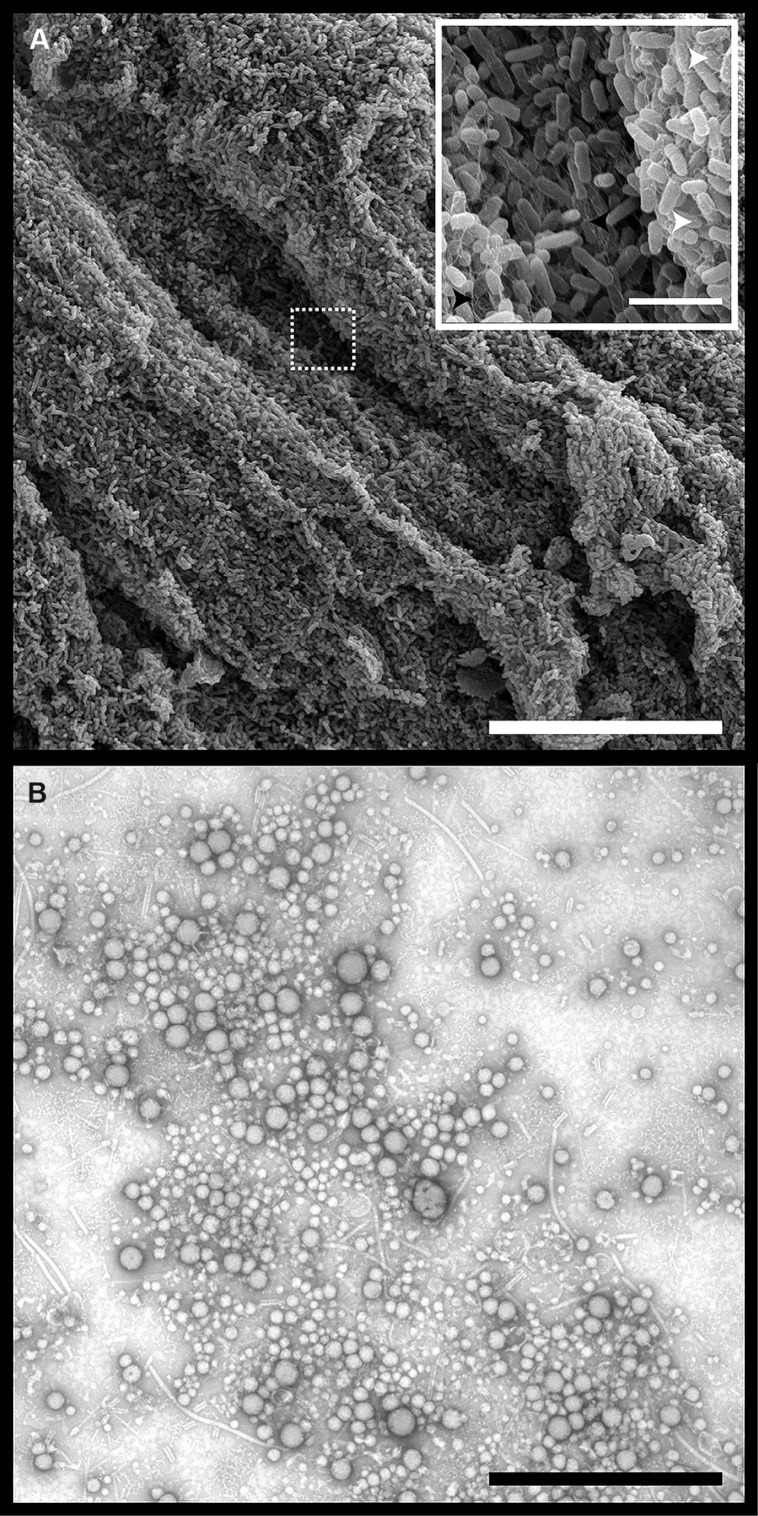
***Pseudomonas aeruginosa* biofilms contain densely packed whole cells, self-produced matrix material, and extracellular outer membrane vesicles (OMVs). (A)** Scanning electron micrographs of *P. aeruginosa* PAO1 grown on tryptic soy agar for 24 h. Dashed box indicates area of magnification. Scale bars represent 30 μm (main) and 3 μm (inset). Inset symbols: White arrowheads indicate OMVs on the surface of whole cells. Black arrowheads indicate dehydrated extracellular matrix material. **(B)** Transmission electron micrograph of negatively stained OMVs purified from a 24 h *P. aeruginosa* biofilm. Scale bar represents 1 μm.

*Pseudomonas aeruginosa* grown on agar for 24, 48, or 96 h were imaged using scanning electron microscopy (SEM). A representative image from the 24 h samples showed evidence of biofilm structures and maturation based on the organization of clusters of individual bacteria connected by a mesh of dehydrated extracellular matrix material (**Figure 2A**). OMVs were observed at the surface of the cells (**Figure 2A**, *inset*), and could be isolated from the CFS of a 24 h biofilm culture (**Figure 2B**). The OMVs were similar to those previously described for PAO1 cultures (Schooling and Beveridge, 2006; Murphy et al., 2014) and were comprised of a single membrane layer and are present in a range of sizes (~20–200 nm).

### BIOFILM WHOLE CELLS AND OMVs SHOW DISTINCT PATTERNS OF INDIVIDUAL PROTEIN ABUNDANCE OVER TIME

**FIGURE 3:**
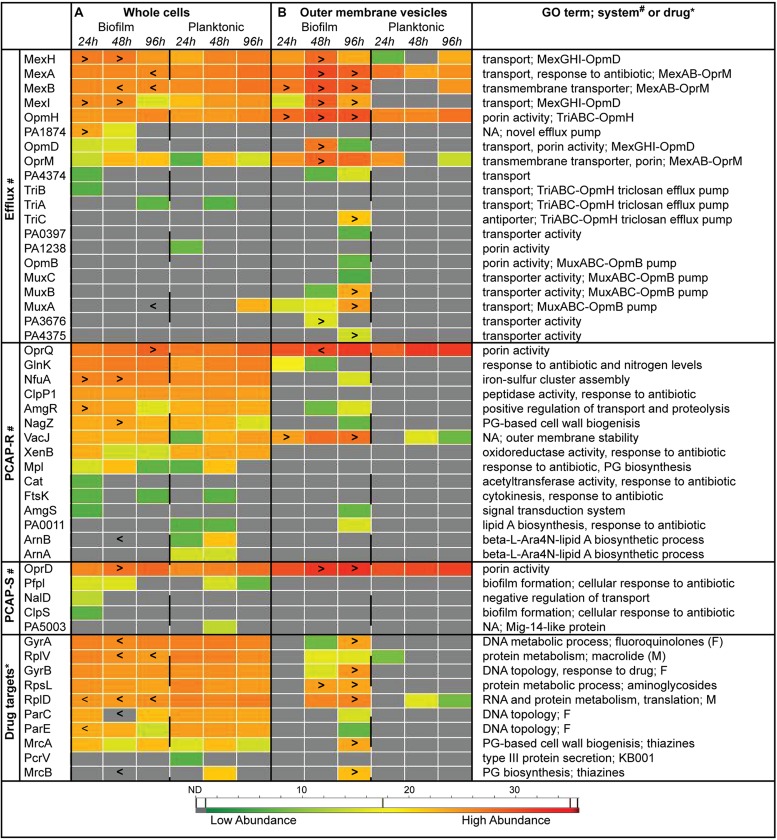
**Individual protein abundance data suggest differential antibiotic resistance strategies in *Pseudomonas aeruginosa* biofilms over time.** Heat maps representing average protein abundance in whole cell **(A)** and OMV **(B)** replicates with warm colors depicting higher mass spectrometry intensity values. Proteins were separated into functional categories relevant to antimicrobial resistance (see “Materials and Methods” for details). Symbols: “>,” biofilm protein abundance is significantly greater than time- and sample type-matched planktonic controls (e.g., 24 h biofilm whole cells vs. 24 h planktonic whole cells; *p* < 0.05); “<,” biofilm protein abundance is significantly less than time- and sample type-matched planktonic controls (*p* < 0.05); * and # denotes categories where a specific system or drug is listed for the protein, respectively. F, fluoroquinolones; M, macrolides; NA, none available; ND, not detected, gray boxes; PCAP-R, PCAP-resistance; PCAP-S, PCAP-susceptibility; PG, peptidoglycan.

Heat maps representing protein abundance show a substantial amount of variation between biofilm and planktonic samples at the individual protein level over the three time points examined (**Figure 3**). Specifically, 14, 24, and 12% of the select group of 50 whole cell proteins had significantly different abundance levels in the 24, 48, and 96 h samples (*p* < 0.05), respectively (**Figure 3A**). Similar numbers of proteins were more (8) or less (10) abundant in biofilms compared to planktonic whole cells. While eﬄux proteins showed a mixed pattern of abundance, the PCAP resistance (PCAP-R) proteins (higher in biofilms) and drug targets (lower in biofilms), were more consistently affected. Intriguingly, the eﬄux group of proteins seemed to be the most affected by the age of the biofilm, with higher levels of eﬄux proteins at 24 h, and lower levels at 96 h (vs. time-matched planktonic controls). Furthermore, eﬄux proteins belonging to the same complex behaved in similar ways. For example, both MexH and MexI of the MexGHI-OpmD pump (Aendekerk et al., 2002) were increased in early biofilm whole cell samples. Similar to whole cells, biofilm and planktonic OMVs showed unique protein abundance patterns with 6, 22, and 32% of the select group of proteins having significantly different abundance levels in the 24, 48, and 96 h samples (*p*< 0.05), respectively (**Figure 3B**). In contrast to the whole cells, the differentially abundant proteins were almost exclusively higher in biofilm OMVs (20/21) across all categories and time points. Overall, fewer proteins were detected in planktonic OMV samples (10 of 50).

### BIOFILM AND PLANKTONIC CULTURES CONTAIN POOLS OF SHARED AND EXCLUSIVE PROTEINS

**FIGURE 4:**
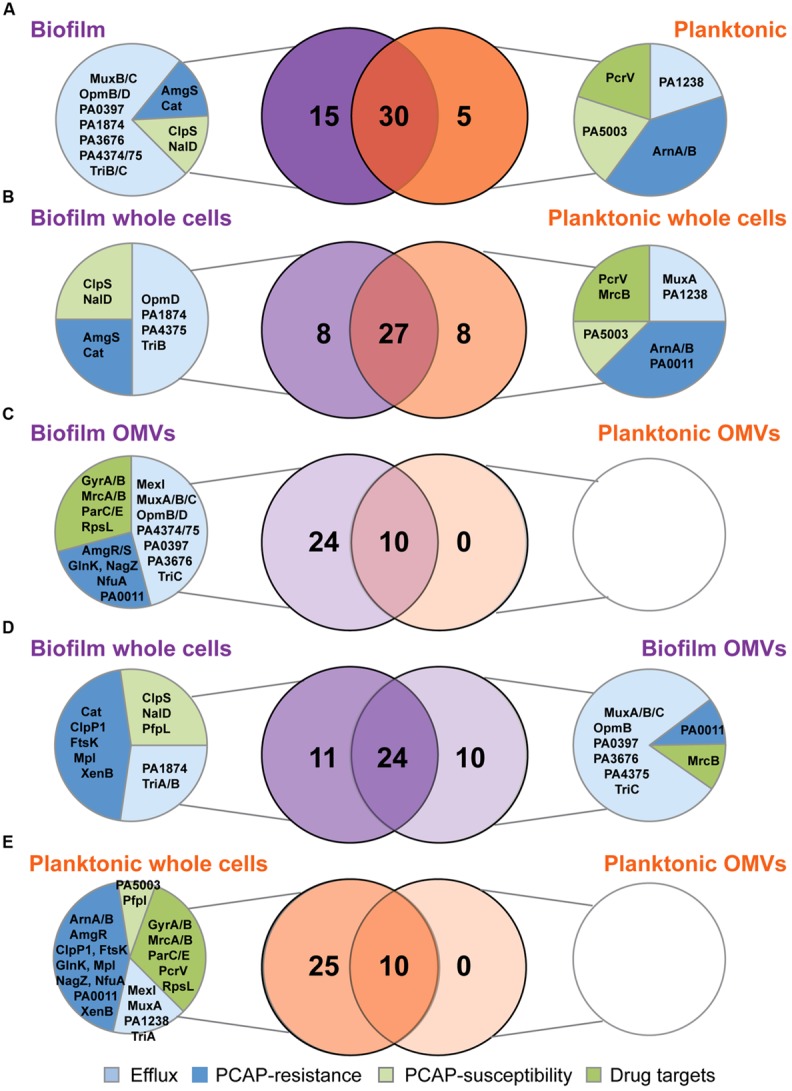
**Individual subproteomes have unique antibiotic and resistance proteins with both redundant and distinct functions. (A–E)** Venn diagrams showing shared and exclusive proteins among growth modes and sample types. Biofilm sample groups are shown in purple and planktonic sample groups are shown in orange. Unique proteins for each comparison (if applicable) are further divided into functional categories with blue pie slices representing proteins with known roles in antimicrobial resistance (eﬄux and PCAP-resistance proteins) and green pie slices representing proteins with known roles in antimicrobial susceptibility (drug targets and PCAP-susceptibility proteins).

Venn diagrams were used to compare the overlapping and unique proteins in the various replicate groups. At the highest level, comparing the distribution of all 50 proteins in biofilm and planktonic samples (whole cells and OMVs combined), 60% of the proteins were detected in both growth modes, with the remaining proportion belonging predominantly to the biofilm samples (30% of the total; **Figure 4A**). The majority of the proteins exclusive to the biofilm samples were eﬄux proteins (11/15), although one specific transport protein, PA1238, was exclusive to planktonic samples. No drug targets were exclusively detected in biofilms; however, the type III secretion protein PcrV (PA1706), which is the target of an engineered human antibody Fab fragment KB001 (Baer et al., 2009), was only detected in planktonic samples. When whole cells sample groups (24 h, 48 h, and 96 h replicates combined) were compared (**Figure 4B**), a similar percent of the total protein profile was shared (~63%), although the remaining exclusive proteins were equally distributed between biofilm and planktonic whole cell samples. Importantly, despite having equal counts of exclusive proteins, the functional distribution of the proteins was not equal. Similar to above, biofilm whole cells had greater numbers of unique eﬄux proteins, and lacked unique drug targets. In direct contrast to the whole cells, the OMV samples (24, 48, and 96 h replicates combined) had a much smaller pool of shared proteins (~30%), and no unique proteins were detected in the planktonic OMVs (**Figure 4C**). Strikingly, 7 of the 24 proteins that were exclusive to biofilm OMVs were drug targets. When whole cells and OMVs were compared (**Figures 4D,E**), major differences were noted: (i) biofilm whole cells and OMVs had a larger pool of shared proteins (24 vs. 10 in planktonic); (ii) biofilm OMVs contained a much higher number of the 50 antibiotic resistance and susceptibility proteins (34 vs. 10 in planktonic OMVs); and (iii) planktonic OMVs did not contain any unique proteins (vs. planktonic whole cell). Furthermore, one specific drug target, penicillin-binding protein 1B (*mrcB*, PA4700), was below the level of detection in biofilm whole cells, but was highly abundant in biofilm OMVs (**Figure 4D**).

### FUNCTIONAL SUMMARIES INDICATE THAT BIOFILM AND PLANKTONIC OMVs ARE MORE DISSIMILAR THAN MATCHED WHOLE CELLS

**FIGURE 5:**
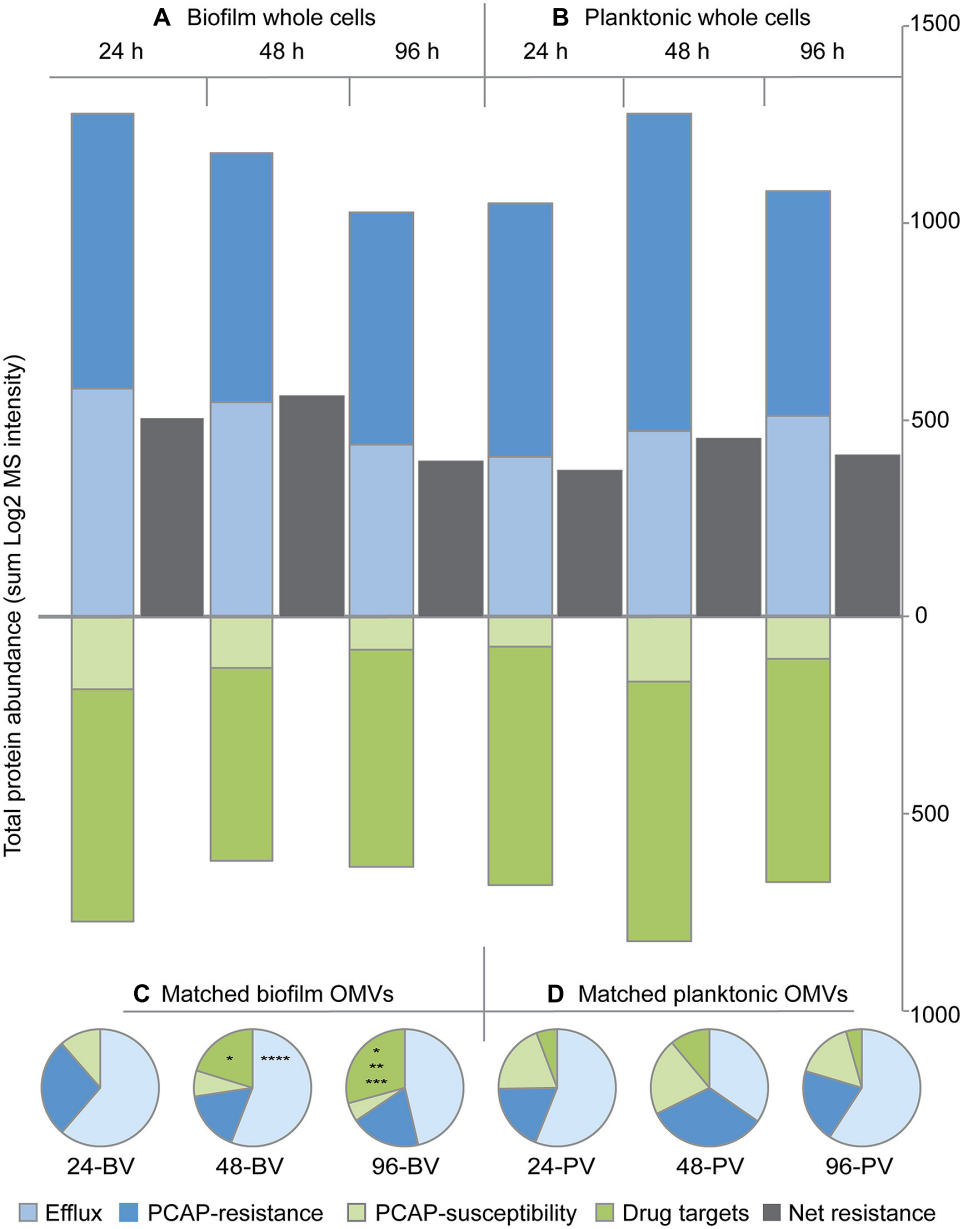
**Protein abundance totals for functional categories indicate that biofilm OMVs are more distinct than biofilm whole cells when compared to their planktonic counterparts.** Bar graphs showing the total protein abundance for biofilm **(A)** and planktonic **(B)** whole cell replicate groups in each of the four functional categories over time. Net whole cell resistance [i.e., (eﬄux + PCAP-resistance proteins) – (drug targets + PCAP susceptibility proteins)] is shown in gray. Pie charts showing total protein abundance for each functional category in matched biofilm **(C)** and planktonic **(D)** OMV samples over time (expressed as a percent of the total abundance). Abbreviations: 24, 24 h; 48, 48 h; 96, 96 h; BV, biofilm OMV; PV, planktonic OMV. Symbols: *p < 0.05 vs. 24-BV drug targets, **p < 0.05 vs. 48-BV drug targets, ***p< 0.05 vs. 96-PV drug targets, *****p < 0.05 vs. 48-PV eﬄux proteins.

Following individual protein analysis, a systems approach was taken, and the total protein abundance in each functional category was compared between replicate groups (**Figure 5**). Interestingly, none of the category totals for whole cells were significantly different when compared between growth modes within time points (i.e., biofilm vs. time-matched planktonic) or between time points within growth modes (e.g., 24 h biofilm whole cells vs. 48 h biofilm whole cells; **Figures 5A,B**). Furthermore, the net resistance estimate (i.e., resistance categories-susceptibility categories) failed to reveal any drastic changes in the whole cell samples (**Figures 5A,B**). In direct contrast, two of the categories showed significant differences in the OMV samples (**Figures 5C,D**). Specifically, at the 48 h time point, the total abundance of eﬄux proteins in biofilm OMVs (48-BV) was statistically greater than time-matched planktonic controls (48-PV; *p* < 0.05). In addition, OMVs harvested from 96 h biofilms (96-BV) were significantly enriched in drug targets compared to 96 h planktonic OMVs (96-PV). This prominent identification of drug targets in biofilm OMVs was also significantly affected by the age of the culture, as the total amount increased as a function of time (**Figure 5C**); specifically, the levels were significantly higher at 48 h compared to 24 h (*p* < 0.05), and significantly higher in 96 h compared to 48 h (*p* < 0.05). No time-dependent changes were detected in total categorical-abundance within the planktonic OMV samples (**Figure 5D**).

## DISCUSSION

A clear demonstration of a biofilm-specific gene expression or protein profile that contributes to adaptive antimicrobial resistance has remained elusive (Drenkard, 2003; Fernandez and Hancock, 2012). This study examined the proteome of the model biofilm former *P. aeruginosa*, and compared the abundance levels of 50 antibiotic resistance and susceptibility proteins (i.e., the resistance subproteome) between biofilm and planktonic cultures at three time points. Herein, we demonstrate the effect of individual proteins vs. systems analysis on the interpretation of proteomic data, and highlight the benefits of each strategy.

Examination of individual whole cell protein quantities over time showed that on average ~16% of the biofilm resistance subproteome was differentially abundant compared to planktonic controls. The size of the effect agrees with early studies that demonstrate a greater response rate using MS-based strategies (Sauer et al., 2002; Southey-Pillig et al., 2005) compared to transcriptomics (Whiteley et al., 2001; Folsom et al., 2010). We show that while PCAP-R proteins and drug targets were consistently altered in biofilm whole cells (increased and decreased, respectively), eﬄux proteins showed heterogeneous patterns depending on the individual protein and time point. Specifically, the proteins MexA and MexB, of the eﬄux pump MexAB-OprM, were significantly lower in biofilm replicates (vs. planktonic) at both 48 h and 96 h (**Figure 3A**). While this may seem counterintuitive considering the prominence of this system in previous antibiotic resistance studies (Li et al., 1995; Srikumar et al., 1999; Morita et al., 2001), these studies were completed under standard laboratory growth conditions (i.e., using planktonic cultures), and may not be applicable to the biofilm conditions. Other have found that both mexAB-oprM and mexCD-oprJ expression is decreased in developed biofilms, and that individual biofilms comprised of *P. aeruginosa* stains lacking MexAB-OprM, MexCD-OprJ, MexEF-OprN, or MexXY did not have altered antibiotic resistance profiles compared to wild type controls (De Kievit et al., 2001). In contrast, the proteins MexG and MexI, of the eﬄux pump MexGHI-OpmD, were present at higher levels in early biofilm samples (**Figure 3A**). This system, first identified in 2002, confers resistance to vanadium (Aendekerk et al., 2002), which has bacteriostatic effect on *P. aeruginosa* cultures under conditions of iron limitation (Baysse et al., 2000). It was later demonstrated that this pump also plays a role in the production of quorum sensing molecules (AHL and PQS), and that the mutation of *mexI* or *opmD* resulted in decreased, rather than increased, resistance to a variety of antimicrobials (Aendekerk, 2005). Consequently, increased abundances in our samples are likely a result of generalized increases in quorum sensing activity in the high-cell density environment of biofilm communities (De Kievit, 2009), and not a biofilm-specific mechanism of resistance. Another protein that was more abundant in biofilms replicates, PA1874, belongs to an eﬄux pump that has previously been shown to be involved in biofilm-specific antibiotic resistance (Zhang and Mah, 2008); the gradual decrease in its abundance over the three time periods (**Figure 3A**) suggest the role of PA1874 may be less prominent in established biofilms.

Additional individual whole cell proteins that were significantly more abundant in biofilms include four of the PCAP-R proteins, namely OprQ (PA2760), NfuA (PA1847), AmgR (PA5200), and NagZ (PA3005; **Figure 3A**). These proteins have diverse GO annotations (Winsor et al., 2010), and were increased at various time points throughout biofilm development; thus, it is likely that they do not constitute a concerted mechanism of adaptive resistance. Interestingly, three proteins in the PCAP-R category with roles in lipid A biosynthesis, specifically PA0011, ArnA, and ArnB, were not detected in biofilms (**Figure 4B**). Modification of the lipid A portion of LPS has been detected in CF isolates of *P. aeruginosa* [as described above (Ernst, 1999; Ernst et al., 2005; Cigana et al., 2009)], can occur in response to environmental stimuli, and is a recognized strategy for evasion of the innate immune response (for review see Trent, 2004). Accordingly, recent studies have reported changes to lipid A structure during *P. aeruginosa* biofilm growth *in vitro* (Ciornei et al., 2010). Although further speculation on the role of these three proteins in biofilm-specific resistance is beyond the scope of this paper, additional MS-based examination of the large collection of proteins involved in LPS biosynthesis and modification (King et al., 2009) during biofilm formation will likely provide invaluable insight.

In contrast to the whole cells, individual OMV proteins were almost universally higher in biofilms as compared to planktonic controls. In addition, as demonstrated in the **Figure 4**, many proteins were unique to biofilms OMVs, and no proteins were exclusively detected in planktonic OMVs (vs. planktonic whole cells or biofilm OMVs). The reason for this cannot be determined with the techniques employed in this study, although, these results suggest that, overall, the biofilm OMV proteome is enriched in the specific proteins captured in this focused analysis of the resistance subproteome. A detailed analysis of the entire OMV proteome is necessary to determine if this is a global phenomenon. The increased detection of both eﬄux proteins and porins in the biofilm OMVs is interesting, especially in light of the recent demonstration that purified MexB proteins are capable of drug-transport in artificial proteoliposomes under an imposed chemical proton gradient (Welch et al., 2010). The independent functioning of transport proteins within OMVs has never been demonstrated.

The examination of individual protein abundances and exclusive pools in both biofilm and planktonic cultures provides valuable information on specific targets that may individually affect the antibiotic resistance. What it fails to capture is the potential functional outcome of the system. Strikingly, when we look at each of the four categories in the resistance subproteome as a whole, we see that none of them are statistically different in the whole cell cultures. This suggests that there may not be one clear-cut, biofilm-specific, protein-based adaptive resistance mechanism in whole cell *P. aeruginosa*. Conversely, it suggests that many subtle changes in the biofilm whole cell proteome may accumulate to collectively attain high levels of resistance. This type of effect has been demonstrated for individual genetic mutations, where the combination of several gene deletions, each with minor effects on resistance, can results in substantial increases in the concentration of antimicrobials that will effectively inhibit growth (El’Garch et al., 2007). Alternatively, a major caveat to our approach is that it does not capture the functional activity of the proteins in our samples, and it is possible that our modeling of the functional outcome of the system is overly simplistic. Combining our current approach with additional functional studies will further improve our understanding of these systems.

What can be taken from the systems-based analysis is the significantly greater abundance of drug targets in the OMV samples. Much attention has been paid to the mutation of targets (e.g., DNA gyrase and/or topoisomerase) in *P. aeruginosa* and their role in fluoroquinolone resistance (for reviews see Neu, 1988; Hooper and Wolfson, 1989; Piddock, 1999; Ruiz, 2003). Our data, however, suggest a separate mechanism involving these proteins that appears to be specific to biofilms, whereby the targets are diminished in the whole cell and concentrated in extracellular structures. The first observation (i.e., decreased whole cell concentrations) is not surprising considering these targets are involved in core metabolic process (Winsor et al., 2010), and biofilms are generally considered to be less metabolically active than their planktonic counterparts. What is unexpected, however, is the identification of 90% of these targets in the biofilm OMVs, and that many are present at abundance levels similar to the biofilm whole cells. Importantly, others have shown the aminoglycoside gentamicin bound to the surface of putative OMVs in the process of formation (Kadurugamuwa et al., 1993); however, the specific target it was attaching to was not identified. One of the proteins we identified, 30S ribosomal protein S12 (*rpsL*, PA4268) is bound by this same class of antibiotics. Importantly, few of these targets appear in planktonic OMVs, and none appear in the 24 h biofilm OMV replicate group, suggesting that this potential mechanism of resistance may be specific to established biofilms. To determine if this is an active process capable of providing biofilm-specific protection from antimicrobials, we require further studies to show specific antimicrobial-drug target interactions in OMVs, and quantifiable contributions to whole cell resistance are required.

In summary, this study demonstrates the benefit of using MS-based strategies to study complex systems. Specifically, we identify a potential biofilm-specific adaptive resistance mechanism outside of the whole cell, which would have escaped detection using transcript-based methods such DNA microarray. Furthermore, we show the effect of individual vs. systems-based analysis on the interpretation of our data, and laid the groundwork for future studies that will elucidate strategies to combat highly recalcitrant microbial biofilms.

## MATERIALS AND METHODS

### BACTERIAL STRAINS, MEDIA, AND REAGENTS

The parental strain for all studies was *P. aeruginosa* PAO1. Planktonic cultures were grown in 400 ml of tryptic soy broth (TSB; BD, Franklin Lakes, NJ, USA) and biofilms were grown on an equal volume of tryptic soy agar (TSA; BD) solidified in a glass dish (190 mm × 100 mm; Corning, Tewksbury, MA, USA). All reagents, unless otherwise stated, were obtained from Sigma-Aldrich Canada Co. (Mississauga, ON, Canada).

### SAMPLE PREPARATION AND PROTEIN QUANTIFICATION

Sterile TSB and TSA were inoculated with 5 ml of overnight culture normalized to an optical density at 600 nm (OD_600_) of 3.0. The inoculum was evenly distributed by gentle agitation (planktonic cultures) or spread over the agar surface with a sterile glass rod (biofilm cultures). Three biological replicates of each sample type were grown statically at 37°C for 24, 48 or 96 h for a total of 36 individual samples. Following incubation, biofilms were scraped off the TSA with a sterile scoopula and suspended in 400 ml of sterile TSB. All samples were normalized to an optical density (OD_600__nm_) of 1.0 before further processing. Whole cells were pelleted by centrifugation (12,000 × *g*, 10 min, 4°C; Avanti J-E, Beckman Coulter, Pasadena, CA, USA), treated with a protease inhibitor (Roche Diagnostics, Indianapolis, IN, USA), and stored at -20°C. The CFS containing the OMVs was retained and processed as previously described (Murphy et al., 2014). Briefly, the CFS was subjected to four rounds of ultracentrifugation (50,000 × *g*, 1.5 h, 4°C; Beckman L8-55M ultracentrifuge, Beckman Coulter). The resulting pellet containing the OMVs was washed and resuspended in Tris-HCl (pH 8.3), filtered through a 0.45 μm cellulose acetate membrane syringe filter (Thermo Scientific, Ottawa, ON, Canada), and then sedimented by centrifugation (21,000 × *g*, 30 min, 4°C; Eppendorf 5424 microcentrifuge, FA-45-24 rotor, Mississauga, ON, Canada). The final pellet was resuspended in 500 μl of Tris-EDTA (pH 8.3), treated with protease inhibitor (Roche Diagnostics), and stored at -20°C until further processing. Prior to enzymatic digestion, whole cell and OMV samples were sonicated on ice (4 × 15 s with 60 s cooling periods, setting 3, Ultrasonic Processor XL, Misonix Inc., Farmingdale, NY, USA). Protein yield from the whole cell and OMV samples was measured using a Micro BCA protein assay kit as per the manufacturer’s instructions (Thermo Fisher Scientific, Waltham, MA, USA).

### PROTEIN DIGESTION

Protein (15 μg) was digested as previously described (Foster et al., 2003). Briefly, proteins were solubilized in a twofold volume of denaturation buffer (6 M urea/2 M thiourea in 10 mM HEPES, pH 8.0), followed by a 30-min treatment with a reduction buffer (10 mM dithiothritol in a 50 mM ammonium bicarbonate [ABC] buffer), and a 20-min treatment with an alkylation buffer (55 mM iodoacetamide in 50 mM ABC). Digestion with 0.3 μg Lys C enzyme per sample (3 h) and 0.3 μg trypsin (16 h; Princeton Separations, Adelphia, NJ, USA) was stopped by adding 40 μl of 0.1% trifluoroacetic acid for every 100 μl of digestion solution. The resulting peptides were desalted and concentrated using MonoSpin^TM^ C18 microcolumns as per the manufacturer’s instructions (GL Sciences, Torrance, CA, USA), and then lyophilized using a speed vacuum concentrator (Savant Instruments, Holbrook, NY, USA). The samples were reconstituted in 0.1% formic acid in water prior to analysis.

### LIQUID CHROMATOGRAPHY-TANDEM MASS SPECTROMETRY (LC-MS/MS)

Liquid chromatography-tandem mass spectrometry (LC-MS/MS) was completed as previously described (Park et al., 2014). Briefly, 5 μl of tryptic peptides were separated using an EASY-nLC 1000 chromatography system (Thermo Fisher Scientific, reverse phase mode, 0.1% formic acid as the mobile phase) paired with an EASY-Spray ES801 column (75 μm × 50 cm) containing PepMap RSLC C18 (2 μm) stationary phase (Thermo Fisher Scientific). Peptides were eluted from the column over 120 min (including a pre-run equilibration and a post-run wash) using a 0 to 30% acetonitrile gradient, at a rate of 250 nL/min and a temperature of 40°C. Eluted peptides were sprayed directly into an EASY-Spray integrated emitter (Thermo Fisher Scientific) for fed nano-electrospray ionization (ESI) using a Q Exactive hybrid quadrupole-Orbitrap mass spectrometer. Specifically, a Q Exactive Orbitrap, a nitrogen-filled higher-energy dissociation (HCD) collision cell, and an Orbitrap mass analyzer were used for parent ion mass measurement, fragmentation, and MS scans, respectively. Finally, spectrum and peak list generation were performed using Q Exactive 2.2 and Xcalibur 2.2 (Thermo Fisher Scientific) with the following acquisition parameters: MS resolution 70,000 FWHM, MS/MS resolution 17,500 FWHM, target 1 × 10^e^6 ions, 10 MS/MS scans/cycle, 15 s dynamic exclusion.

### DATA ANALYSIS

Raw data files were extracted and searched against the UniProtKB *P. aeruginosa* ATCC15692 database (5564 entries) using MaxQuant (Cox and Mann, 2008) quantitative proteomics software (version 1.4.0.5, Max Planck Institute of Biochemistry, Martinsried, Germany) with the following settings: label-free quantification (LFQ), Trypsin/P digestion, maximum of two missed cleavages and five modifications per peptide, 0.02 Da fragment ion mass tolerance, and 20.0 PPM parent ion tolerance. Variable peptide modification included deamidation of asparagine and glutamine plus oxidation of methionine. Fixed peptide modifications included carbamidomethyl alkylation of cysteine. Modifications used in protein quantification included acetylation of the n-terminus, and oxidation of methionine. A false discovery rate (FDR) of 1% was applied for both peptides and proteins using decoys generated with a reverse ATCC15692 database. MaxQuant results were loaded into Perseus (Max Planck Institute of Biochemistry) and the LFQ intensity values were transformed [log_2_(x)] for downstream bioinformatics (heat maps and Venn diagrams) and statistics. Statistical analysis for individual proteins (**Figure 3**) was completed in Perseus by comparing growth modes (i.e., biofilm vs. planktonic) within sample type (i.e., whole cell and OMV) and time point replicate groups using two-sample Student’s *t*-test. A permutation-based FDR, based on 250 randomizations of the data, was used to control the type I error rate. Statistical analysis of the functional categories (**Figure 5**) was completed using Prism (GraphPad Software Inc., CA, USA). Specifically, a one-way analysis of variance (ANOVA) with a Tukey’s post-test was used to compare the total protein abundance within functional groups between replicate groups. Alpha was set to 0.05 for both the *t*-test and the ANOVA. Additional analysis and graphical representation of the data was completed using Microsoft Excel (Microsoft Corporation, WA, USA). The information provided herein is compliant with the Minimum Information about a Proteomics Experiment (MIAPE) Mass Spectrometry Informatics (MIAPE-MSI) guidelines (Binz et al., 2008).

### PROTEIN ANNOTATIONS

Proteins were manually annotated using various methods. Briefly, “eﬄux” proteins were identified by performing an advanced search of the Pseudomonas Genome Database (Winsor et al., 2010) by selecting “*Pseudomonas aeruginosa* PAO1,” search field “name (protein/product),” term “eﬄux.” This search returned 39 results; 15 of these proteins were detected in our samples. Three additional porins, OpmD (Aendekerk et al., 2002), OpmH (Mima et al., 2007), and OpmB (Mima et al., 2009) were manually identified as belonging to multidrug eﬄux systems. “Resistance” and “susceptibility” proteins were identified using the “browse by *Pseudomonas aeruginosa* PAO1 genome project function class” function, found under the simple search menu of the database (Winsor et al., 2010). This search returned 70 results; 28 of these proteins were detected in our samples. Individual proteins were separated into either resistance or susceptibility based on transposon mutant screens (Breidenstein et al., 2008; Alvarez-Ortega et al., 2010; Fernandez et al., 2012), or individual studies as indicated in Table S1 in Supplementary Materials. Seven of the proteins identified in this search, PA1238 (alternately known as OpmJ), PA1874 (Zhang and Mah, 2008), OprM, MexA, MexB, TriA, and TriB were allocated to the “eﬄux” protein group. Finally, protein drug targets were identified using the browse by “Drugs and their targets” function, found under the simple search menu of the database (Winsor et al., 2010). This search returned 12 results (excluding the eight 16S and 23S rRNAs); 10 of these proteins were detected in our samples.

### IMAGING

Biofilms specimens and OMVs were prepared for SEM and transmission electron microscopy (TEM), respectively, as previously described (Murphy et al., 2014). Briefly, sections of TSA and overlying biofilm were fixed in 2% glutaraldehyde (30 min) and osmium tetraoxide (30 min; Canemco Inc., Canton de Gore, QC, Canada). Samples were dehydrated in a series of ethanol solutions (50–100%), critical point dried, and then coated with 15 nm gold using a K550X sputter coater (Emitech Ltd., Kent, UK). Images were acquired with a S-570 scanning electron microscope (Hitachi High Technologies Canada, Inc., Toronto, ON, Canada). OMVs were whole mounted on carbon-coated 200-mesh copper grids (Gilder Grids Ltd., Lincolnshire, England), and negatively stained with 1% uranyl acetate. Images were acquired with a CM-10 transmission electron microscope (Phillips Innovation Services, Eindhoven, Netherlands), paired with a Morada 11-megapixel charge-coupled device (CCD) camera (Olympus Soft Imaging Solutions, Munster, Germany).

## Conflict of Interest Statement

The authors declare that the research was conducted in the absence of any commercial or financial relationships that could be construed as a potential conflict of interest.
