# Supplementary material for: Antimicrobial targets localize to the extracellular vesicle-associated proteome of Pseudomonas aeruginosa grown in a biofilm
Source: Front Microbiol. 2014 Sep 3;5:464. doi: 10.3389/fmicb.2014.00464 (PMC4153316; doi:10.3389/fmicb.2014.00464)
Supplement: Supplementary file 1 [file Table_1.PDF]

Supplementary Table 1. Resistance and susceptibility references for Figure 3

PA	Protein	Category	Reference
2760	OprQ	PCAP-R	(Arhin and Boucher, 2010)
5288	GlnK	PCAP-R	(Alvarez-Ortega et al., 2010)
1874	NfuA	PCAP-R	(Daung-nkern et al., 2010)
1801	ClpP1	PCAP-R	(Fernandez et al., 2012), (Breidenstein et al., 2008)
5200	AmgR	PCAP-R	(Lee et al., 2009)
3005	NagZ	PCAP-R	(Asgarali et al., 2009)
2800	VacJ	PCAP-R	(Shen et al., 2012)
4356	XenB	PCAP-R	(Blehert et al., 1999)
4020	Mpl	PCAP-R	(Tsutsumi et al., 2013)
0706	Cat	PCAP-R	(Beaman et al., 1998)
2615	FtsK	PCAP-R	(Alvarez-Ortega et al., 2010), (Breidenstein et al., 2008)
5199	AmgS	PCAP-R	(Lee et al., 2009)
0011	PA0011	PCAP-R	(Alvarez-Ortega et al., 2010), (Shen et al., 2012)
3552	ArnB	PCAP-R	(Breazeale et al., 2005)
3554	ArnA	PCAP-R	(Breazeale et al., 2005)
0958	OprD	PCAP-S	(Li et al., 2012)
0355	Pfpl	PCAP-S	(Fernandez et al., 2012), (Breidenstein et al., 2008)
3574	NalD	PCAP-S	(Breidenstein et al., 2008), (Sobel et al., 2005)
2621	ClpS	PCAP-S	(Alvarez-Ortega et al., 2010), (Fernandez et al., 2012)
5003	PA5003	PCAP-S	(Alvarez-Ortega et al., 2010)

Alvarez-Ortega, C., Wiegand, I., Olivares, J., Hancock, R. E. W., and Martinez, J. L. (2010). Genetic Determinants Involved in the Susceptibility of Pseudomonas aeruginosa to β-Lactam Antibiotics. *Antimicrobial Agents and Chemotherapy* 54, 4159–4167.

Arhin, A., and Boucher, C. (2010). The outer membrane protein OprQ and adherence of *Pseudomonas aeruginosa* to human fibronectin. *Microbiology* 156, 1415–1423.

Asgarali, A., Stubbs, K. A., Oliver, A., Vocadlo, D. J., and Mark, B. L. (2009). Inactivation of the Glycoside Hydrolase NagZ Attenuates Antipseudomonal β - Lactam Resistance in *Pseudomonas aeruginosa*. *Antimicrobial Agents and Chemotherapy* 53, 2274–2282.

Beaman, T. W., Sugantino, M., and Roderick, S. L. (1998). Structure of the hexapeptide xenobiotic acetyltransferase from *Pseudomonas aeruginosa*. *Biochemistry* 37, 6689–6696.

Blehert, D. S., Fox, B. G., and Chambliss, G. H. (1999). Cloning and sequence analysis of two *Pseudomonas* flavoprotein xenobiotic reductases. *Journal of Bacteriology* 181, 6254–6263.

Breazeale, S. D., Ribeiro, A. A., McClerren, A. L., and Raetz, C. R. H. (2005). A formyltransferase required for polymyxin resistance in *Escherichia coli* and the modification of lipid a with 4-amino-4-deoxy-l-arabinose: identification and function of udp-4-deoxy-4-formamido-l-arabinose. *Journal of Biological Chemistry* 280, 14154–14167.

Breidenstein, E. B. M., Khaira, B. K., Wiegand, I., Overhage, J., and Hancock, R. E. W. (2008). Complex Ciprofloxacin Resistome Revealed by Screening a *Pseudomonas aeruginosa* Mutant Library for Altered Susceptibility. *Antimicrobial Agents and Chemotherapy* 52, 4486–4491.

Daung-nkern, J., Vattanaviboon, P., and Mongkolsuk, S. (2010). Inactivation of nfuA enhances susceptibility of *Pseudomonas aeruginosa* to fluoroquinolone antibiotics. *J Antimicrob Chemother* 65, 1831–1832.

Fernandez, L., Breidenstein, E. B. M., Song, D., and Hancock, R. E. W. (2012). Role of intracellular proteases in the antibiotic resistance, motility, and biofilm formation of *Pseudomonas aeruginosa*. *Antimicrobial Agents and Chemotherapy* 56, 1128–1132.

Lee, S., Hinz, A., Bauerle, E., Angermeyer, A., Juhaszova, K., Kaneko, Y., Singh, P. K., and Manoil, C. (2009). Targeting a bacterial stress response to enhance antibiotic action. *Proc. Natl. Acad. Sci. U.S.A.* 106, 14570–14575.

Li, H., Luo, Y.-F., Williams, B. J., Blackwell, T. S., and Xie, C.-M. (2012). Structure and function of OprD protein in *Pseudomonas aeruginosa*: From antibiotic resistance to novel therapies. *International Journal of Medical Microbiology* 302, 63–68.

Shen, L., Ma, Y., and Liang, H. (2012). Characterization of a novel gene related to antibiotic susceptibility in Pseudomonas aeruginosa. *J Antibiot (Tokyo)* 65, 59–65.

Sobel, M. L., Hocquet, D., Cao, L., Plesiat, P., and Poole, K. (2005). Mutations in PA3574 (nalD) Lead to Increased MexAB-OprM Expression and Multidrug Resistance in Laboratory and Clinical Isolates of *Pseudomonas aeruginosa*. *Antimicrobial Agents and Chemotherapy* 49, 1782–1786.

Tsutsumi, Y., Tomita, H., and Tanimoto, K. (2013). Identification of Novel Genes Responsible for Overexpression of ampC in *Pseudomonas aeruginosa* PAO1. *Antimicrobial Agents and Chemotherapy* 57, 5987–5993.
